# Diaspora linkages benefit both sides: a single partnership experience

**DOI:** 10.1080/16549716.2019.1645558

**Published:** 2019-07-31

**Authors:** Beverley Kramer, Roy Zent

**Affiliations:** a School of Anatomical Sciences, Faculty of Health Sciences, University of the Witwatersrand, Johannesburg, South Africa; b Division of Nephrology and Hypertension, Department of Medicine, Nashville, TN, USA; c Department of Cell and Developmental Biology, Vanderbilt University Medical Center, Nashville, TN, USA; d Veterans Affairs Hospital, Nashville, TN, USA

**Keywords:** Alumni, diaspora, health research, capacity development, resource constrained countries

## Abstract

The emigration of physicians and scientists from resource-constrained countries decreases the country’s ability to undertake research. Re-establishing research environments and increasing capacity reduced by these losses are important, particularly in the health sciences. One mechanism for re-establishing strong health sciences research is the introduction of an Alumni Diaspora Fellowship Programme. We define the beneficial effects of a successful single partnership in an Alumni Diaspora Programme. This Host/Alumnus collaboration demonstrates that bi-directional advantages have accrued for both the Host Institution situated in a resource-constrained country and the Alumni’s Institution, located in a high-income country. In addition to expanding research in the resource-constrained country, collaborations expanded to other faculty beyond the Alumnus in the sending Institution, in multiple fields including those not readily available in the high-income country (HIV, TB, malaria). The environment at the host Institution in the resource-constrained country has been enriched by increased research publications, training of young scholars (over 200 trained in manuscript and grant application writing), and substantial advances in biomedical informatics. There has been considerable knowledge exchange and development between both Institutions, showing that ‘brain circulation’ and Diaspora Programmes are valuable strategies for expanding research.

## Background

The burden of disease in the resource-constrained world continues unabated despite major advances in medical and scientific knowledge. One possible reason for this is that insufficient biomedical research is performed in these areas. The USA and Europe are the leaders in publishing in biomedical research [1–4], while Africa has fallen markedly behind in its contribution to global knowledge. Over the last few decades, researchers in Africa have contributed low numbers of articles [5] and the impact has decreased since the 1990s [6].

One reason for the marked decline in publications during the 1990s was the massive emigration of physicians and scientists from resource- constrained countries. The reasons for the mass emigration of professionals from Africa are multiple. Quality of life, access to advanced technologies, higher salaries [7] and differences in remuneration and living conditions in low-income countries [8], career and educational opportunities in centres of knowledge [9], poor working conditions [10], political upheaval and instability [8] are some of the reasons cited for the ‘brain drain’ from Africa.

The loss of health professionals from southern Africa [10,11], while of value to the recipient country, is detrimental to the health-care system from which relocation has occurred. As skilled health professionals leave, those who remain behind must contend with greater workloads and declining job satisfaction [10]. This relocation not only impacts the health-care system, but also the ability of those remaining to undertake research [12]. The exodus of professionals from Africa could, however, be harnessed to the advantage of both the high income and resource-constrained world by developing programs that ‘bring back’ alumni to their home institutions, i.e. ‘brain gain’, to reinvigorate those areas depleted by ‘brain drain’. This concept is extensively explored in the literature [7–10,13–19], where the positive value of Diaspora programs was shown to stimulate endogenous capacity development, research outputs and skills development, as well as having an impact on the development of policies by health agencies and governments [10,13].

The necessity to improve research outputs and increase capacity development was recognized in the Faculty of Health Sciences, University of the Witwatersrand, in Johannesburg, South Africa in early 2008. This culminated in the initiation of a number of important strategies [20] to build a rich scientific environment for the future. While a specific theory or model of capacity development was not identified [see 21], we ascribe to the World Health Organization definition of capacity development i.e. ‘the development of knowledge, skills, commitment, structures, systems, and leadership to enable effective health promotion’ [22]. One of these strategies, an Alumni Diaspora Fellowship Program, took advantage of a large number of alumni lost to the country over many years through political and economic conditions [18].

## University of the Witwatersrand (Wits) Alumni Diaspora Program

The Wits Alumni Diaspora Program was initiated in 2010 to address key challenges in the institution, and to encourage alumni with strong research experience to ‘return home’ for short periods of time [18]. From 2010 to 2013, 22 alumni from Europe, New Zealand, the USA and Canada returned on brief research Fellowships as part of an internally funded program. In 2013, the Carnegie Corporation of New York invested in the Program which not only enabled alumni to return home for brief periods of time, but also allowed for a return visit of a Wits researcher to the resource-rich environment of the alumnus. Between the years 2014–2018 a further 24 alumni visits to Wits took place, allowing for the development of networks and collaborations. Each Alumnus/a visit is funded by a budget of approximately $4500.

## Initiation of the Wits-Vanderbilt collaboration

One of the first Fellowships initiated in early 2010, was between Wits and an alumnus based in the USA at the Vanderbilt University Medical Centre. This collaboration has grown slowly, but significantly, into a strong program that has provided benefits to research and capacity development, both in South Africa and the USA. It is an excellent example demonstrating how Diaspora programs can be highly successful for both institutions.

The first visit by the alumnus from Vanderbilt Medical School to the Wits Faculty of Health Sciences took place in 2010 and has continued annually to the present (2019). The alumnus was supported by a co-facilitator, also from Vanderbilt, on most of the trips. Both the alumnus and the co-facilitator are senior internationally recognized independently funded researchers. The alumnus is a board-certified practising medical specialist. They both have significant experience with grant writing and reviewing for numerous organizations including the National Institute of Health (NIH) as well as editorial experience with high impact scientific journals. In addition, senior content experts from Vanderbilt have travelled to South Africa on different occasions with the purpose of setting up specific relationships between the institutions.

The initial visit by the alumnus was aimed at providing scientific and grant application writing workshops. However, over the years, the Wits-Vanderbilt Program expanded into a multifaceted program, as the Wits host and alumnus teamed potential collaborators to Wits researchers prior to each visit. We present here the following highlights of some of the major accomplishments of the program.

### Scientific and grant application writing

One major achievement of the program is the development of a scientific writing course that has encapsulated the preparation of both grants and manuscripts (Table 1). This was implemented in 2010 and was delivered by the Vanderbilt alumnus and the co-facilitator. The program has two distinct components; didactic lectures on grant application and manuscript writing as well as small group sessions where the attendees present their grants and also review other people’s grants so as to get the perspective from the reviewer’s side. The ‘small group’ sessions, of approximately 12 participants per group, were aimed at training and honing the skills of both junior and mid-level investigators. The skills gained were passed on to other staff members and postgraduate students in their domains. Between 2010 and 2018, over 215 individuals have been trained in manuscript and grant application writing. These workshops have been extremely well-received by the attendees, who claim they assisted with the attainment of major grants as well as publication of several articles from Wits based researchers.

**Table 1.** Outcomes of Wits-Vanderbilt single partnership experience showing the development of capacity.

		Activity	Deliverable	‘Skill’ enhanced
1.	Scientific and grant application writing courses	Over 215 participants in delivered courses	Numerous scientific articles and grants submitted Manuscripts accepted. Some of the grants have been awarded.	Scientific writing Grant application writing Communication Teamwork
2.	Education and training	Administrative processes	Initiated processes to ease functioning of medically qualified staff in both countries due to restrictions in Health Council rules	Examples of fields which have been opened to access: Molecular pathology Disease s of lifestyle
3.	Medical Informatics	Introduction of REDCap™	Training of Wits individual by Vanderbilt Initiation of biomedical informatics system at Wits Resulted in over 3300 active user accounts on REDCap Initiation of a hospital medical records system in one sector Training of and collaboration with African counterparts. Spread to local NGOs	Move from file-based to electronic collection of data Faster analysis of data on projects Expansion of Wits biostatistics systems in order to train for and handle large amounts of data [23] Over 65 publications utilizing REDCap™ Over 28 postgraduate dissertations utilizing REDCap™
4.	Research collaborations		Expansion of the number of research projects beyond that of the original Host:Fellow relationship	Knowledge exchange Knowledge enhancement to both institutions Addition to scientific knowledge through numerous publications Communication Contribution to alleviation of disease Understanding of African burden of disease
5.	Return visits	Multiple visits between senior staff of institutions	Identification and enabling of systems to facilitate collaboration of various individuals across multiple fields of research	Building trust between institutions Identification of key research areas

### Education and training

One of the major goals of the Wits-Vanderbilt alliance was to improve accessibility for training of faculty and postgraduate students at both institutions. This was put into place by the alumnus and the two administrations who removed as many administrative barriers as possible for these interactions to occur (Table 1). This facilitated travel by faculty between the institutions, which has resulted in major collaborations. These are discussed in detail below. In addition, they have led to the exposure of both trainees and faculty to very different clinical scenarios in diverse environments. For example, Wits clinicians were exposed to state-of-the-art molecular pathology, while Vanderbilt clinicians were exposed to the burden of disease ‘experience’ in Africa.

### Medical informatics

One of the early projects formulated between the two institutions revolved around developing the field of biomedical informatics at the Wits Faculty of Health Sciences, where the major part of clinical data resides in paper-based files. In this context, Vanderbilt extended an invitation to a Wits faculty member to become trained in this field (Table 1). This training took place in 2013 and was organized in such a way that the Wits faculty member was able to join already organized graduate didactic seminars at Vanderbilt Medical Center. In addition, the Wits faculty member spent 4 months at Vanderbilt where he was integrated within the informatics department. He was introduced to a diverse array of bioinformatics tools including REDCap™, an open-source software package developed by Vanderbilt Medical Center. This software was introduced to the Wits Faculty of Health Sciences (Table 1) and led to a substantial impact on research. The process of setting up this software was published, in order to assist other African institutions in attaining in-house Information Technology [24]. The software is now available throughout Africa, where it is used extensively for clinical studies and other applications that require data capture.

By December 2013 there were over 150 active users and more than 300 projects created on REDCap™ at Wits. By 2018 this had grown to over 3300 user accounts, of which 1300 are currently active (Mare, personal communication). There are 2700 projects, 945 of which are in production and 1440 are still in development. The rest of the accounts are inactive or archived. On average, 650 different users log-in to REDCap™ daily. In addition, hands-on training workshops are provided by a Wits REDCap™ administrator and have been central to the increased numbers of users. Approximately 65 published articles and 28 postgraduate dissertations from the Wits FHS have thus far utilized the REDCap™ platform for data collection (Table 1).

With the help and participation of the Director, Office of Research Informatics at Vanderbilt University, an African Consortium was initiated by Wits in 2014. Wits has developed the capacity to act as the support node for the African REDCap™ consortium partners in the South Africa Standard Time (SAST) zone. A REDCap™ Africa day was hosted in 2016 drawing more than 110 participants of the consortium from countries across Africa countries such as Uganda, Cameroon and Senegal as well as participants from a host of local institutions. A similar event was held again held in 2017 and 2019, primarily for Wits faculty, staff and students.

The impact of this medical informatics initiative has also been spread to other non-governmental organizations in Johannesburg, including the Aurum Institute (a public benefit, non-profit organization based in Johannesburg, South Africa associated with Wits) (Table 1). The Vanderbilt alumnus has coordinated interactions between his institute and Aurum such that there has been a flow of staff between the two. Vanderbilt has actively helped with the setting up of medical informatics at Aurum. The long-term plan with this interaction is to set up an all- African data exchange that will actively tackle the problems of medical informatics within the African environment. A tripartite collaborative data management strategy between the Wits Faculty of Health Sciences, the Aurum Institute and Vanderbilt Medical University was initiated in 2018 which will work towards improving data science within the African continent.

In addition to the introduction of RedCap™ to South Africa, the Wits faculty member also acquired the skills to set up hospital medical records that can be used for research purposes.

The interactions between Wits, Aurum and Vanderbilt, in the information science space, have continued to grow with multiple visits between the institutions. The relationship has allowed for Vanderbilt Medical Center to provide significant input into how this field can be expanded on the African continent in many different fields including data capture and interpretation. Importantly, the benefits have accrued to all three participating institutions; Vanderbilt has been provided with research opportunities by the vast amounts of data that are generated by researchers at Wits and Aurum.

### Research collaborations

Over time, the Wits Host and alumnus introduced various researchers in the Wits Faculty of Health Sciences to researchers at Vanderbilt Medical Center, which led to the initiation of several collaborations between the institutions (Table 1). This has been important for enhancing research in both organizations. While the alumnus was able to introduce new technological advances found in the high-income country, South Africa was able to provide a unique resource as its population has a massive disease burden in a population group which differs from that of the USA. Importantly a healthy relationship was established that was based on a 50:50 partnership. A dictum for the relationship was ‘Research with, rather than in or about Africa, is the goal’ [25]. In addition, the research was relevant to the local environment and mutually beneficial in the furtherance of knowledge.

The Wits-Vanderbilt collaborations have grown beyond the initial alumnus-Wits Host relationship to include non-Wits alumni at Vanderbilt with matched researchers at Wits. The areas of research range from infectious diseases such as tuberculosis and malaria to cardio-metabolic diseases and basic health sciences. All these collaborations have been investigator initiated after the introduction of collaborators from both institutions. The number of these collaborative projects has been increasing over time and are based on the premise that they are equal partnerships. As such, joint appointments for investigators from both institutions have been established and some of these investigators are splitting their time and effort between the organizations. So far, these collaborations have led to 12 publications in international journals between 2011 and 2017, oral presentations at international congresses, and joint funding, including joint Wits-Vanderbilt grants from the NIH.

### Return visits

Two return visits of the Wits Host to Vanderbilt ensued. The Host was accompanied on these visits by the Wits Dean of the Faculty of Health Sciences and other senior academic members of staff. Interactions between the Deans of the two institutions led to a solidifying of aims and an understanding of the needs of the two institutions. Recently, a delegation comprising the Vice Chancellor of Wits and Wits Faculty of Health Sciences senior management visited Vanderbilt University to strengthen the relationships between Vanderbilt and Wits. The group identified further potential strategic collaborations and research opportunities which will be leveraged by both institutions.

## Discussion/conclusion

While Africa had fallen markedly behind in its contribution to global knowledge, this trend may be reversing as sub-Saharan Africa (SSA) has significantly increased its quantity of peer–reviewed research articles (including reviews and conference proceedings) at the rate of 8.5% per annum between 2003 and 2012. Even so, this rate is less than two comparator countries, Malaysia and Vietnam, who grew even faster [26]. SSA’s share of global research has increased from 0.44% to 0.72% from 2003 to 2012. However, South Africa only increased from 0.07% to 0.09% [26]. In South Africa, health sciences comprised the highest percentage of the total article output [26] and South Africa is now the highest research output-producing country in Africa [27].

A major reason for this drop in productivity was the massive emigration of physicians and scientists from resource-constrained countries during the 1990s. Over this period 56% of all migrating physicians originated from the under-resourced world and some medical research institutions in sub-Saharan Africa including Nigeria were forced to close due to loss of highly skilled health professionals [12]. An excellent example of specific loss to South Africa is that it contributed 7% of the foreign-trained UK physician workforce [12]. A deficit in highly skilled health professionals from Africa was recognized by the United Nations as one of the ‘greatest obstacles to Africa’s development’ [28].

While Amienyl [27] maintains that the infrastructure and finances at African Universities require strengthening in order to attract academics back from the diaspora, there are alternative strategies whereby this can be achieved. Whitworth et al. [29], Whitworth et al. [30] and Sewankambo et al. [31] have described requirements and existing initiatives to strengthen capacity for health research in Africa and emphasize the urgent need to build the next generation of African scientists. Sewankambo et al. [31] describe a collaborative program between an institute in a low-income country and an institute from a high-income country which has resulted in the graduation of numerous PhDs and also substantially increased peer-reviewed articles, thus showing the benefits of this type of collaborative venture. In addition, as the Wits Program has been run on a relatively small budget, similar Programs to develop research capacity could be duplicated in other low- and middle-income countries.

The fundamental idea of Diaspora programs is to foster collaborations between scientists from the rich and under-resourced parts of the world. This is a mechanism for strengthening research in the under-resourced areas of the world, but also offers many opportunities to rich countries to enhance and increase their research. The diaspora scientists can contribute in a variety of ways to developing capacity and skills, and through these collaborations, local institutions can evolve into sustainable centres of excellence [18].

The Wits-Vanderbilt single-partnership study of a Diaspora Program supporting collaboration between an alumnus and his Institution based in the USA of America and a Host and her Institution based in Africa is an example of a program whose strategy was to bring back alumni for short periods of time in order to strengthen research capacity and expand existing research niches and collaborations as was the case by Sewankambo et al. [31]. In the current partnership, the African health institution has provided interesting research opportunities through large patient databases and unique diseases not see in the western world, while the institution based in the USA has provided expertise and training in their areas of strength. As a result, the program has expanded over time and now includes numerous researchers at both the Host and Fellows institution.

Temporary ‘brain circulation’ does not only provide benefits to the home institution, but to the alumnus’ Institution as well. In this single-partnership study, this has occurred through the expansion of research into fields not readily available in the high-income country at the alumnus’ institution, while at the host institution there have been contributions to both capacity development and growth. Thus, the benefits of the program are bidirectional. In addition, over time, incorporation of researchers other than the alumnus from the alumnus’ home institution has expanded collaborations and enabled the growth of research at both institutions. Advantages to the alumnus’ institution have accrued through research initiatives in areas of HIV, TB and maternal and child health not seen in the well-resourced setting. The greatest strength of the program is a net gain in knowledge exchange and development to both institutions.

Brain circulation as supported by Diaspora programs has been proposed to be benefit institutions in a number of ways. Data indicate (Hunter, 2013) that scholars who circulate outside of their own countries are more productive than those who do not. Furthermore, harnessing of Diaspora scientists by governments has supported input and strengthening of national research agendas [14]. Saravia and Miranda [9] suggest that the way in which to redirect ‘brain circulation’ is by creating opportunities at home. The Wits-Vanderbilt collaboration has shown that this strategy is possible and can result in meaningful, highly impactful relationships between institutions.

While this single-partnership study may be a ‘drop in the ocean’ [16] in developing research collaborations in resource-deprived countries, it is part of a much wider Carnegie-Wits Alumni Diaspora Programme. Other Wits seeded Host-Fellow relationships are growing and expanding. These collaborative associations are one way of ‘repatriating’ alumni. The Pew Latin American Fellowship is another way of ensuring scientific growth in resource-deprived countries [16].

Recently, Saint-Blancat [19], an Italian alumna declared**: ‘ …** I’m strongly motivated to do anything I can to give back to my country a part of all that it gave to me … but I have never found the way.’ The Carnegie-Wits Alumni Diaspora program has provided a mechanism for alumni to ‘give back’ to their alma mater and in so doing, has expanded research and capacity development.

In conclusion, the Wits-Vanderbilt collaboration nested in the larger Carnegie-Wits Diaspora Alumni Programme has resulted in an expansion of research projects particularly in HIV, TB and maternal health at both the host and Fellow’s institutions. Furthermore, this collaboration has developed scientific and grant application writing skills at Wits, which has occasioned publications and approved grants, markedly improved the electronic database system for collection of data and patient records through the introduction of REDCap™ and resulted in knowledge exchange and development at both instiutions. Thus, the Diaspora Programme has proven to be beneficial for the collaborating institutions in both the resource-constrained and high-income countries.

## Paper context

The manuscript describes the research and capacity development benefits accrued to two health sciences institutions, one in a high-income country and the other in a resource-constrained country through a collaboration which utilizes Diaspora Alumni linkages. The Diaspora linkage program which was initiated between the two institutions in 2010 has resulted in research skills development and expansion of research areas for both institutions. Knowledge exchange and development will contribute towards building the next generation of African Health Scientists.
